# Attitudes and Determinants of Mandatory Vaccination against COVID-19 among the General Population of Cyprus: A Nationwide Cross-Sectional Study

**DOI:** 10.3390/vaccines10030438

**Published:** 2022-03-13

**Authors:** Konstantinos Giannakou, Maria Kyprianidou, Alexandros Heraclides

**Affiliations:** Department of Health Sciences, School of Sciences, European University Cyprus, Nicosia 1516, Cyprus; m.kyprianidou@external.euc.ac.cy (M.K.); a.heraclides@euc.ac.cy (A.H.)

**Keywords:** vaccination, COVID-19, attitudes, mandatory vaccines, obligatory, SARS-CoV-2, Cyprus

## Abstract

Vaccinations for the prevention of coronavirus disease (COVID-19) are important to control the ongoing pandemic. A much-discussed strategy to increase vaccination coverage is mandatory vaccination; however, its legitimacy and effectiveness as a measure are doubtful. This study aims to investigate the attitudes of the general population of Cyprus towards COVID-19 mandatory vaccination and to identify the factors influencing individuals’ attitudes towards such policy. An online cross-sectional study was conducted, using a self-administered, anonymous questionnaire to collect information on sociodemographic and health-related characteristics, trust, and satisfaction about the healthcare system and utilization of preventive healthcare services, COVID-19 vaccination information, general vaccination knowledge, and attitudes towards mandatory vaccination. A total of 2140 participants completed the survey, with 27.8% being in favor of mandatory vaccination. We found that as the age increases by one year, the odds of supporting mandatory vaccination increase by 1.04 units (OR 1.04, 95% CI: 1.02–1.05). In addition, those who reported increased trust in national healthcare authorities’ guidelines and recommendations (OR 3.74, 95% CI: 3.11–4.49) and those satisfied with the healthcare system (OR 1.38, 95% CI: 1.16–1.65) and follow doctor’s instructions (OR 1.29, 95% CI: 1.03–1.61), were significantly more likely to support mandatory vaccination while those who had underage children living in the household were significantly less likely to support mandatory vaccination (OR 0.69, 95% CI: 0.50–0.94). Public health authorities need to develop well-organized vaccination campaigns in which accurate evidence-based information would be disseminated with respect to individuals’ autonomy.

## 1. Introduction

At the end of December 2019, Chinese public health authorities reported several cases of acute respiratory syndrome in Wuhan City, China. The new zoonotic disease, now referred to as coronavirus disease 2019 (COVID-19) caused by severe acute respiratory syndrome coronavirus 2 (SARS-CoV-2), spread rapidly in practically all parts of the world [1]. The global death toll of the COVID-19 pandemic has been estimated to 5.5 million over the first two years of its existence [2], with long-standing illness and long-term complications affecting several survivors [3,4]. Additionally, the COVID-19 pandemic has resulted in an unprecedented impact in practically all aspects of life, including international production and trade, with a subsequent global economic impact [5].

Over the first year of the COVID-19 pandemic, the lack of effective vaccines resulted in other means of emergency containment measures, including lockdowns and strict imposition of personal protective measures (e.g., mandatory mask use). Since the end of 2020, however, the licensing of vaccines against SARS-CoV-2 was a reality. The first COVID-19 vaccines were based on a novel mRNA-based technology [6] (e.g., Pfizer-BioNTech’s mRNA BNT162b2 (Pfizer-BioNTech, Pfizer Inc., New York City, NY, USA, and BioNTech SE, Mainz, Germany) and Moderna’s mRNA-1273 (Moderna, Cambridge, MA, USA)) and next-generation viral vector technology [7] (e.g., Oxford-AstraZeneca’s ChAdOx1/AZD1222 (Oxford/AstraZeneca, Oxford, UK)). Following these early vaccines, several more have been licensed using similar (e.g., Johnson & Johnson’s INJ-7843735/Ad26.COV2.s ((Janssen/Johnson & Johnson, Beerse/ New Brunswick, NJ, Belgium/USA), Gamaleya’s Sputnik V (Gamaleya Research Institute, Moscow, Russia)) or other technologies (e.g., Whole-Pathogen Inactivated virus technology such as SinoVac’s CoronaVac (Sinovac Biotech, Beijing, China), and Protein Subunit technology, such as Novavax’s NVX-CoV2373 (Novavax, Gaithersburg, MD, USA)) [8].

Collectively, the efficacy of COVID-19 vaccines in preventing severe infection and death in infected individuals ranges from about 80% (adenovirus-vectored COVID-19 vaccines) to over 95% (mRNA-based vaccines) [9]. Post-vaccination studies have revealed that the effectiveness of the above vaccines starts waning among vaccinated individuals, after a period of about three months [10], which renders a booster dose necessary for containment of the spread globally [11]. As regards safety, a meta-analysis of all clinical trials conducted on the main COVID-19 vaccines between 2020–2021, concluded that the proportion of vaccinated individuals who experienced extreme adverse effects was remarkably low [9].

Despite the overwhelming evidence on the effectiveness and safety of the aforementioned COVID-19 vaccines, a huge global pro-vaccination campaign was required in order to convince the world population to receive a free vaccination regime against the disease. Despite this unprecedented international awareness campaign, more than a year after the release of effective COVID-19 vaccines, a substantial proportion (about 40%) of the global population remained unvaccinated [12]. In EU/EEA countries, this proportion was lower (about 25%) [13], but still large enough to compromise attempts for the total control and containment of the pandemic. In an attempt to overcome the aforementioned COVID-19 vaccine hesitancy, several countries have considered and even implemented in some cases, strict vaccine mandates. For example, since February 2022, Austria became the first European country to enforce a law making vaccination against COVID-19 compulsory for all citizens over 14 years of age [14], while several other countries have introduced vaccination mandates for specific population sub-groups, such as healthcare providers [15,16,17], public sector employees and employees working in large companies [14,18], school staff, police officers/firefighters/soldiers, and the elderly [14]. In the Republic of Cyprus, where the current study was conducted, an intense vaccination campaign resulted in 60% of adults being fully vaccinated against COVID-19 (slightly lower than the EU/EEA average) [13], yet no vaccine mandates had been implemented at the time of publication of the current article, as the general public has strongly opposed such approaches and remains highly skeptical regarding COVID-19 vaccination [19].

Such opposition has also been observed in other countries, as vaccine mandates have generally been received with skepticism and even outrage by specific population groups, while the ethical aspects of such approaches have been widely debated [20,21,22,23,24,25,26,27,28]. Although vaccine mandates might appear to be a reasonable measure for increasing vaccination uptake [29], understanding and respecting the public’s perceptions on this issue, is essential in planning effective measures for increasing vaccination uptake and avoiding fueling vaccine hesitancy, as well as introducing inequality among citizens, specific employees, and other population sub-groups, as noted in some countries throughout Europe [30,31,32]. The current cross-sectional study aimed to investigate attitudes towards, as well as the determinants, of mandatory vaccination against COVID-19, among a nationwide sample from the Republic of Cyprus.

## 2. Materials and Methods

### 2.1. Study Design, Population, and Data Collection

This was a nationwide cross-sectional online survey. This study was reported following the Strengthening the Reporting of Observational Studies in Epidemiology [33]. The referent population included Greek-Cypriot men and women aged 18 years old and above, living in the five government-controlled municipalities of the Republic of Cyprus (Nicosia, Limassol, Larnaca, Paphos and Ammochostos). Data collection took place during 15 November 2021 and 7 January 2022. A nonprobability convenience sampling approach was used to recruit participants using an online self-administered questionnaire. This convenience sampling strategy was inevitable, due to the quarantine restrictions imposed by the on-going COVID-19 pandemic, which consequently influenced sampling possibilities. The questionnaire was administered using Google Forms and dispersed using instant messaging apps (e.g., WhatsApp, Viber), social media platforms (e.g., Facebook, Instagram), and social networking sites (e.g., LinkedIn). To assess for potential selection bias, we compared our sample characteristics with statistics of the general population in Cyprus. Overall, we managed to keep a similar proportion of the study population among the five government-controlled districts of the Republic of Cyprus [Nicosia (47%), Limassol (25%), Larnaca (15%), Paphos (7%), and Ammochostos (7%)] (*p*-value > 0.05) [34].

### 2.2. Questionnaire

The questionnaire contained 47 open-ended and closed-ended questions in the Greek language covering a wide range of potential determinants, including sociodemographic characteristics (e.g., age, gender, educational level, etc.), health-related characteristics and information regarding trust, satisfaction with the healthcare system and utilization of preventive healthcare services (e.g., presence of chronic diseases, use of preventive healthcare services etc.), information related to COVID-19 vaccination, sources of vaccines information, and participants’ general vaccine knowledge and attitudes towards mandatory vaccination. The questionnaire was developed by our research team, based on our previous research experience and extensive literature search [35,36,37,38,39,40,41]. Prior to the actual study, face validity was tested in a small pilot study involving 50 participants to assess the clarity and the applicability of all items of the survey, as well as to address wording problems. The pilot sample was not included in the study sample.

#### 2.2.1. Sociodemographic Characteristics

Age was provided in years and sex was recorded as men or women. Educational level was classified in three categories: (i) up to secondary education (participants who completed up to high school); (ii) undergraduate education (participants who had a college or a university degree); (iii) postgraduate education (participants who had a master or a PhD degree). Marital status was recorded as never married, married/in cohabitation, or separated/divorced/widowed. Occupation was recorded as private or state employee, freelance, student, unemployed, housewife, or retired. Healthcare professional occupation was evaluated using a binary question (Yes vs. No). Annual income was classified as: (i) low (≤€6500); (ii) moderate (€6,500–19,500); and (iii) high (>€19,500). Underage children living in the household was evaluated using a binary question (Yes vs. No).

#### 2.2.2. Participants’ Health Status and Attitudes towards Healthcare Services

The presence of chronic diseases was evaluated using a binary question “Chronic diseases (at least one)” (Yes vs. No). Use of preventive healthcare services, trust in official guidelines and recommendations of the official healthcare authorities, satisfaction with the healthcare system and following doctor’s instructions/medical adherence were evaluated on a Likert scale ranging from 1: Not at all, No trust, No satisfied, Not at all, to 5: Very often, Very strong trust, Extremely satisfied and Very often, respectively. The Cronbach’s α-value for internal reliability for this section was 0.69.

#### 2.2.3. Participants’ Attitudes towards Mandatory COVID-19 Vaccination

Mandatory vaccination status was categorized as a binary variable using the question “Do you think that COVID-19 vaccination should be mandatory on the general population” (Yes vs. No) and on a Likert scale ranging from 1: Strongly disagree to 5: Strongly agree. The remaining items assessing attitudes towards mandatory COVID-19 vaccination were evaluated on a Likert scale ranging from 1: Strongly disagree to 5: Strongly agree. The Cronbach’s α-value for internal reliability for this section showed strong internal consistency (α = 0.96).

#### 2.2.4. Information about Participants’ COVID-19 Vaccination Status

Vaccination status was evaluated using a binary variable (Yes vs. No). There were questions about the number of doses and the type of COVID-19 vaccine received. Intention to receive another dose if requested and belief that vaccine helped to prevent COVID-19 disease were evaluated on a Likert scale ranging from 1: Not at all to 5: Very much. In addition, there were questions about the intention if they have not received the COVID-19 vaccine to date, if they plan to receive it, and if they belong to a vulnerable group (diabetic, immunosuppressed, etc.) to whom vaccination is recommended (‘I do not know’, ‘Yes’ and ‘No’). The sources of information about vaccination were defined using the question “Which are the main sources of information on COVID-19 vaccination? Choose all that apply” with possible answers internet/social media, TV/newspapers/radio, scientific journals, personal doctor, colleagues/friends/family, conferences/seminars, and the option other. In addition, the reasons for vaccination were defined using the question “For which of the following reasons you were vaccinated? Choose all that apply” with possible answers to protect myself, to protect my family, to protect others, because of my job, and the option to report other reason. Finally, the reasons for hesitating to get vaccinated against COVID-19 were defined using the question “For which of the following reasons were you not vaccinated? Choose all that apply” with possible answers fear of adverse side effects, expedited development and approval of the vaccine, concerns about getting infected from the vaccine, not liking needles, not belonging to a vulnerable group, not thinking that COVID-19 is dangerous to my health, preference for natural immunity, waiting for other people to get vaccinated, and the option to report another reason.

#### 2.2.5. General Vaccination Knowledge

To test participants’ general knowledge towards vaccination, we used a questionnaire, from which a 12-item scale is derived. We included questions concerning controversial subjects that are often related to vaccinations such as alleged links to autism and allergies, and whether vaccinations can be replaced by antibiotics. Participants were asked to indicate whether the item was correct or not, by choosing from “True”, “False”, and “I do not know”. General vaccination knowledge items showed acceptable internal consistency (α = 0.70).

### 2.3. Ethics Approval

This study was conducted according to the Declaration of Helsinki guidelines and approved by the Cyprus National Bioethics Committee (CNBC) (ΕΕΒΚ ΕΠ 2021.01.219). Participation was anonymous, and all the participants were informed about the study aim and objectives before participating. Before completing the questionnaire, the participants gave their consent by answering a “Yes/No” question on a mandatory electronic informed consent form that included statements regarding voluntary participation and anonymity.

### 2.4. Statistical Analysis

The distribution of the continuous variables was examined using Shapiro–Wilk normality test. Participants’ characteristics are presented as mean ± standard deviation (SD) for continuous measures with normal distribution, while categorical variables are presented as absolute (n) and relative (%) frequencies. The chi-square test of independence was used to evaluate any association between mandatory COVID-19 vaccination (categorical variable) and the categorical participant characteristics. The Student’s *t*-test was used for the comparison of mandatory COVID-19 vaccination (categorical variable) and continuous baseline participant characteristics with normal distribution.

Participants’ vaccination knowledge was measured by 12 questions with three possible answers (‘True’, ‘False’, and ‘I do not know’). A vaccination knowledge score was created for each participant by scoring the individual knowledge question items, giving a score of 1 for each question correctly answered and 0 for each question answered incorrectly or in case of lack of knowledge (i.e., answered “I do not know”). We calculated the knowledge score of the participants by adding the points of each of the 12 knowledge items (maximum score 12). The tertiles of vaccination knowledge score were defined as follows: low vaccination knowledge score (score ≤ 5), moderate vaccination knowledge score (score 6–8), and high vaccination knowledge score (score ≥ 9). Higher scores indicate a higher vaccination knowledge.

Hierarchical logistic regression analysis was used to examine the association between sociodemographic (Model 1), vaccination knowledge score (Model 2), and presence of chronic diseases, participants’ health status and attitudes towards healthcare services (Model 3) on mandatory vaccination support (Yes vs. No). Firstly, we added the sociodemographic characteristics (gender, age, and occupation, and marital status, underage children living in the household, education, and annual income) as independent variables in a model including mandatory vaccination support (Yes vs. No) as a dependent variable. Then, we added the vaccination knowledge score, and finally the presence of chronic diseases and attitudes towards healthcare services were included in the model. Multicollinearity of the independent variables was checked using the variance inflation factor with all estimates being under 2.9, indicating no multicollinearity between independent variables. Additionally, a correlation matrix was constructed, and all correlation coefficients between independent variables were lower than 0.65.

Radar graphs were constructed to present the reasons for vaccination and the reasons for hesitating to get vaccinated against COVID-19. All statistical tests performed were two-sided with the statistical significance level set at α = 0.05. Statistical analysis was conducted using STATA 14.0 (Stata Corp, College Station, TX, USA) and Microsoft Excel 2013.

## 3. Results

There were a total of 2140 participants among whom 1323 (61.8%) were women. Among the respondents, 594 (27.8%) were in favor of mandatory COVID-19 vaccination and 1539 (72.2%) were opposed to such policy. The mean age of the participants was 38 ± 11 years. The majority of the participants were private employees (n = 1032, 48.8%) and state employees (n = 474, 22.4%). Most of the participants (n = 1472, 69.7%) were married/in cohabitation and about 53% (n = 1133) had underage children living in the household. In addition, most of the participants had completed undergraduate education (n = 495, 45.4%) and earned an annual income of more than 19,500 euros (n = 943, 45.3%) (Table 1).

### 3.1. Participants’ Characteristics by Mandatory Vaccination Support

The mean age of participants was 37 ± 10 years for those who did not support the mandatory vaccination and 41 ± 12 years for those who supported the mandatory vaccination (*p* < 0.001). The largest differences between occupation and mandatory vaccination support were identified in unemployed individuals (74.5 vs. 25.5%, for no and yes, respectively) (*p* < 0.001). In addition, the largest percentage of participant who supported the mandatory COVID-19 vaccination was observed among retired individuals (n = 30, 62.5%) (*p* < 0.001).

Regarding the education level, the largest percentage not supporting the mandatory vaccination was identified in individuals who completed up to secondary education (n = 326, 77.8%), while the largest percentages who support mandatory vaccination were identified among participants who had a postgraduate degree (n = 243, 33.2%) (*p* < 0.001). Moreover, among those who had an annual income more than 19,500 euros, almost 35% supported the mandatory vaccination, while the corresponding percentages among those with a low income or moderate income was 25.3 and 21.9%, respectively (*p* < 0.001). We did not find any statistically significant differences in gender, geographical area, and marital status between mandatory vaccination support groups (*p* > 0.05) (Table 1).

About 18% of the study participants had at least one chronic disease (Table 2). The majority of the participants use the preventive healthcare services moderately (n = 713, 33.4%), do not trust the official guidelines and recommendations by the national healthcare authorities (n = 619, 29.1%), are moderately satisfied with the healthcare system (n = 792, 37.2%), and often follow the doctor’s instructions (n = 1097, 51.4%). We reported statistically significant associations for all attitudes towards healthcare services by mandatory vaccination support (*p* < 0.001). Specifically, the largest percentages of the individuals who support the COVID-19 mandatory vaccination were identified among those who very often use preventive healthcare services (38.7%), have a very strong trust in the official guidelines and recommendations by the national healthcare authorities (80.9%), are extremely satisfied with the healthcare system (75.5%), and follow doctor’s instructions very often (44.9%) (Table 2).

### 3.2. COVID-19 Vaccination Status and Mandatory COVID-19 Vaccination

More than half of the participants were vaccinated against COVID-19 (n = 1143, 54%) (Table S1). The majority of those individuals were vaccinated with two doses (n = 760, 65.6%) and with Pfizer vaccine (n = 792, 68.3%). In addition, most of the participants believe that vaccination helped them prevent COVID-19 infection a lot (n = 384, 28.4%) and the majority did not belong to a vulnerable group (n = 1764, 84.3%). More information regarding participants’ COVID-19 vaccination status overall and by mandatory vaccination support are presented in Table S1.

The most common reasons for vaccination among study participants were to protect themselves (31.1%), to protect their family (30.0%), and to protect others (25.8%) (Figure S1). Moreover, the most common reasons for hesitating to get vaccinated against COVID-19 were due to the expedited development and approval of the vaccine (24.4%), fear of adverse side effects/safety of vaccine (24.2%), and preference for natural immunity (17.6%) (Figure S1). The majority of the participants used internet/social media (31.6%) to be informed about COVID-19 vaccination, followed by TV/newspapers/radio (19.4%) and scientific journals (17.2%).

About 70% (n = 1539) of the study participants do not support mandatory COVID-19 vaccination with more than 50% strongly disagreeing with a mandatory COVID-19 vaccination policy (Table 3). Most of the participants strongly disagree that mandatory disposal of COVID-19 vaccines is ethically and scientifically justified (n = 966, 45.2%), strongly agree that mandatory COVID-19 vaccination of is a policy directed against individual’s personal freedoms (n = 1003, 47.1%), and strongly agree that mandatory COVID-19 vaccination violates human rights (n = 1094, 51.2%). In addition, a substantial proportion of participants strongly disagree that mandatory COVID-19 vaccination is a policy that will reinforce their perception that the COVID-19 vaccine is necessary (n = 982, 46.1%), strongly disagree that mandatory COVID-19 vaccination is a policy that reinforces their perception that vaccine side effects are rare (n = 900, 42.6%), and strongly disagree that mandatory COVID-19 vaccination is a policy that will reinforce their perception that the vaccine has been studied well (n = 946, 44.5%). Also, a substantial proportion of study participants strongly disagree that COVID-19 vaccination should be mandatory for healthcare professionals (n = 748, 35%); however, almost 30% of participants strongly agree with mandatory vaccination of healthcare professionals (Table 3). As expected, we found statistically significant differences among all attitudes towards mandatory COVID-19 vaccination by comparing the two mandatory vaccination support groups (*p* < 0.001).

### 3.3. General Vaccination Knowledge Score

The mean vaccination knowledge score was 6.5, which indicates a moderate vaccination knowledge among the adult general population of Cyprus. Among those who support mandatory vaccination, the mean vaccination knowledge score was eight, whereas among those who did not support mandatory vaccination this was six (Table 4). We reported statistically a significant association between vaccination knowledge level and mandatory COVID-19 vaccination groups (*p* < 0.001). Specifically, the majority of the participants reported as having a moderate vaccination knowledge level (n = 1182, 55.2%) and among them 26% (n = 309) support the mandatory vaccination. Among the participants who had a low vaccination knowledge score (n = 451, 21.1%), only 5% (n = 23) support the mandatory vaccination, while the corresponding percentage among the 507 (23.7%) participants with a high vaccination knowledge score (n = 507, 23.7%) was almost 52%. More information regarding participants’ general vaccination knowledge items is presented in Table S2.

### 3.4. Determinants of Mandatory Vaccination Support

In order to identify independent determinants of mandatory vaccination support, hierarchical logistic regression modeling was applied (Table 5). Firstly, we applied a model adding various sociodemographic characteristics (Model 1). We found that females had 1.26 times higher probability of supporting the mandatory vaccination compared to males (95% CI: 1.01–1.57). In addition, increased age was associated with a higher probability of supporting the mandatory vaccination (OR: 1.04, 95% CI: 1.02–1.05). Students had 3.74 (95% CI: 2.11–6.62) times higher probability of supporting the mandatory vaccination compared to private employees. In addition, divorced/separated/widowed individuals and those with underage children living in the household had 40% (95% CI: 0.39–0.92) and 38% (95% CI: 0.49–0.78) lower probability of supporting mandatory vaccination compared to married/in cohabitation individuals and those who did not have children living in the household, respectively. In addition, individuals who completed postgraduate studies (OR = 1.83, 95% CI: 1.31–2.56) and those with a high annual income (OR = 2.38, 95% CI: 1.44–3.96) had higher probability of accepting mandatory vaccination compared to those who complete up to secondary education and those with a low income, respectively.

When vaccination knowledge score was added in the model (Model 2), we found that the association for age (*p* < 0.001), being a student (*p* = 0.007), and having underage children living in the household (*p* < 0.001) with mandatory vaccination remained statistically significant. The associations for income and particularly educational attainment were attenuated, however, indicating the presence of possible mediation by vaccination knowledge in the association between education/income and mandatory vaccination support. Moreover, we note that for every one unit increase in vaccination knowledge score, the probability of supporting COVID-19 mandatory vaccination increases by 1.50 times (95% CI: 1.42–1.59).

Next, we included participants’ attitudes towards national healthcare services (Model 3). We observed that increased age (*p* < 0.001), having underage children living in the household (*p* = 0.018), and vaccination knowledge score (*p* < 0.001) remained statistically significant. In addition, we reported that increased trust in official guidelines and recommendations by the national healthcare authorities (OR: 3.74, 95% CI: 3.11–4.49), increased satisfaction with the healthcare system (OR: 1.38, 95% CI: 1.16–1.65) and medical adherence (OR: 1.29, 95% CI: 1.03–1.61) increases the probability of supporting COVID-19 mandatory vaccination (Table 5).

## 4. Discussion

This study aimed to investigate the attitudes of the general population of the Republic of Cyprus towards mandatory vaccination against COVID-19 and to identify the main factors influencing individuals’ attitudes towards such policy. We observe that among the respondents, only 27.8% were in favor of mandatory COVID-19 vaccination and 72.2% were opposed. According to our findings, the strongest predictors of mandatory vaccination support were older age, underage children resided in the household, general vaccination-related knowledge score, increased trust in official healthcare authorities’ guidelines, satisfaction with the healthcare system, and medical adherence. Together these results provide important insights for tailor-made interventions by the applicable health policy bodies to inform the general public as to the benefits of vaccination and tackle the public’s concerns in order to boost vaccination uptake.

Our results suggest that more than two-thirds (72.2%) of the general population of Cyprus were opposed to a mandatory COVID-19 policy. More than half of the population deemed that this policy was directed against an individual’s personal freedoms, violates human rights, and that it was ethically and scientifically unjustified. This signifies that a legal obligation for mandatory vaccination may be required and/or be necessary under certain conditions. However, it should be stressed that, before the start of the global vaccination campaigns, approximately 70% of the population in high income countries were somewhat favorable to get vaccinated [42,43,44]. This finding of opposition to mandatory vaccination could be related both to the observed decline in trust in the national healthcare authorities and the government of the Republic of Cyprus during the pandemic as well as due to the spread of misinformation of COVID-19 vaccines efficacy and safety that appeared since the beginning of the pandemic. In fact, most of the participants reported internet and social media (31.6%) as the main sources of information about COVID-19 vaccination.

To date, few studies have examined the public opinion towards mandatory vaccination against COVID-19. In line with our results, a recent study among the general public in Jordan, Kuwait, and other Arab countries showed a lower rate of acceptance to mandatory COVID-19 vaccination (18.4%) [45]. In contrast, our study showed a higher proportion of opponents to COVID-19 mandatory policy, when compared to other similar studies in Germany (51%) [46], United States of America (USA) (44.9%) [47], France (41.9%) [48], Greece (14.8 and 25.7%) [36,49], and Australia (9%) [50]. Of interest, data from a serial cross-sectional survey in Germany showed that support for mandatory vaccination was lowest before the first vaccines against COVID-19 were approved by the European Medicine Agency (21 December 2020) and increased afterwards, indicating that people are more likely to reject new vaccines than established ones [51]. The differences between our findings and those from other studies could be attributed to many factors including actual differences in attitudes towards mandatory vaccination between different countries. Culturally, the Cypriot population is likely particularly sensitive to issues involving mandates of all sorts, probably resulting from long periods of oppression and occupation through its history, with freedom of choice being of paramount importance [52]. Additionally, diverse data collection periods among studies, baseline heterogeneity between the populations studied, as well as strong controversy and polarization between those in favor and those opposed to vaccination mandates, could provide explanations for the differences observed. In addition, due to the online nature of participant recruitment, the sample of our study was relatively younger than that of previous studies and based on the current as well as previous findings [46,48], older age is associated with more favorable pro-mandate attitudes.

Furthermore, our analyses revealed certain characteristics of the population to be associated with mandatory vaccination support. One of the strongest predictors of mandatory vaccination support was age, whereas older age individuals were more likely to support a COVID-19 vaccination mandate for the general population. This finding is consistent with other studies in European countries [46,48,51]. The higher probability among older individuals to support mandatory vaccination is anticipated as this is the most susceptible group and, therefore, most prone in their self-interest to a mandatory COVID-19 policy. In addition, we observed that the presence of underage children in the household was associated with lower probability of supporting COVID-19 mandatory vaccination. This finding could be related with the parents’ acceptance of childhood COVID-19 vaccination, which could accordingly adapt their vaccination perception and attitudes towards a mandatory vaccination program. In fact, the results of a recent cross-sectional study among nurses and midwives in Cyprus identified negative attitudes towards the vaccination of their children with the COVID-19 vaccine [53].

Undoubtedly, mandatory COVID-19 vaccination for the general population is a highly politicized issue. With safety concerns raising the hesitancy levels for COVID-19 vaccines, we found that public trust in official guidelines and recommendations by the national healthcare authorities as well as satisfaction with the national healthcare system were associated with a greater opposition to a mandatory COVID-19 vaccination policy. Lack of trust in the government during the pandemic was associated with greater opposition to a mandatory COVID-19 vaccination policy in France [48], whilst trust in the state and the healthcare authorities was associated with support towards mandatory vaccination in Greece [36,49]. Not surprisingly, the individual’s adherence to doctors’ instructions was associated with support for mandatory COVID-19 vaccination. A possible explanation is that individuals who adhere to doctors’ recommendations at a higher rate, this might influence an individual’s self-protective behavior against COVID-19 as well as affect behavioral intentions and perceptions. These findings highlight the influential role of healthcare professionals and authorities in addressing and mitigating vaccine misconceptions that may be concerning to the public by effectively communicating accurate information via community outreach efforts.

We also sought to determine whether the participants’ degree of knowledge regarding vaccination plays a role in their views concerning a possible mandatory vaccination policy against COVID-19. Our findings revealed that a pro-mandate attitude was significantly associated with increased general vaccination-related knowledge score, even after controlling for various socio-demographics factors and other potential confounders. In other words, a better vaccination knowledge status (i.e., better vaccination knowledge could lead to decreased misconceptions and/or less personal attitudes guiding the vaccine decisions) was associated with a greater probability of supporting a mandatory vaccination. It is noteworthy that previous studies found vaccine-related knowledge to be a predictor of vaccination intention, suggesting that the better the understanding of vaccination, the more likely people might choose to be vaccinated [54,55,56,57,58]. Thus, we assume that health literacy and vaccination awareness could influence the individuals’ attitudes and lead to an increased understanding of the usefulness of vaccination programs in addressing this and future pandemics. This is reflected on the support of the public towards a mandatory vaccination program policy, as they may consider it as crucial and necessary once the benefits are clearly communicated. Interestingly, our study also revealed vaccination knowledge to be a mediator in the association between education and mandatory vaccination support, suggesting behavior, attitudes, and beliefs of the population are formed as part of their education and, therefore, may be predictive of their attitudes.

The COVID-19 mandatory vaccination is a complex politicized issue that entails citizens’ rights and decision-making autonomy. While mandatory vaccination policies have been previously proven successful in achieving high vaccination rates [59,60], a possible mandatory vaccination policy against COVID-19 could be counterproductive, especially if it is not acceptable by a great majority of the population [61]. This could eventually be detrimental to the overall acceptance of vaccines by enhancing suspicion of COVID-19 vaccines, leading to distrust of the national public health authorities. Even when faced with serious public health challenges (e.g., exhaustion of populations and economies affected by serial outbreaks over a long period [28]), and the expected positive effects and impact to society is essential, rigorous criteria must be applied to any consideration of a COVID-19 vaccine mandate. Alternative strategies such as the combination of effective communication, educational, and promotional strategies focused on the efficacy and safety of vaccines could be effective in increasing vaccination coverage. Vaccination against COVID-19 could be considered to be made mandatory only for certain groups of people (e.g., healthcare professionals), only after time has conclusively shown that sufficient numbers of individuals have not actually been vaccinated. In this regard, in this paper, we are not recommending a policy of mandatory vaccination against COVID-19, but merely investigating the attitudes of the population of Cyprus towards it. Although, it is beyond the scope of this study to evaluate the ethical issues related to the implementation of mandatory vaccination, our findings could help public health authorities and policymakers in Cyprus to improve the understanding of the determinants that impact populations’ opinions on mandatory vaccination. This could yield to the development of future communication strategies and educational interventions designed at changing beliefs and motivations towards vaccination, which could lead to the control the spread of infectious diseases, including COVID-19.

To the best of our knowledge, this is the largest survey to date that assesses attitudes towards and determinants of mandatory vaccination against COVID-19, among a nationwide sample from the Republic of Cyprus. Due to the large sample size with national coverage, the study had more than adequate statistical power to detect even small differences among subgroups. Yet, despite this study’s novelty and its potential significance, it has some limitations. This was a cross-sectional design, therefore, causal inferences between predictors and outcomes cannot be made. Moreover, as this study was based on voluntary, self-reported data, we cannot rule out some other sources of bias, inherent in surveys, such as social desirability and selection bias. Data collection was done using a convenience sampling method, promoted through various online channels, such as social media platforms, limiting our study representativeness and the generalizability of the study results. Although internet-based surveys are cost-effective and fast, we cannot exclude the possibility that individuals without access to technologies are probably underrepresented in our sample, whilst certain sub-groups such as individuals exhibiting proactive attitudes about the assessed topic may be largely oversampled, impairing the overall reliability of the study. Also, the response rate for our online survey was not possible to be calculated, since there is no way to ascertain how many individuals might have seen the survey or its links but declined to participate. In addition, our study assessed the general knowledge about vaccination, but not the specific knowledge about COVID-19 vaccines. Whether there are differences in these two types of knowledge is unknown. Future research could explore the association between COVID-19 vaccination-related knowledge and attitudes towards mandatory COVID-19 vaccination. Lastly, these findings are relevant only to the population of Cyprus and thus cannot be extrapolated to other countries.

## 5. Conclusions

To our knowledge, this is the first study that provides insights regarding the attitudes towards, as well as the determinants of mandatory vaccination against COVID-19, among a nationwide sample from the Republic of Cyprus. This study found that more than two-thirds of the study population were opposed toward a mandatory COVID-19 policy, possibly demonstrating that this policy is perceived as directed against their personal freedoms and their human rights. We also found that older age, underage children resided in the household, general vaccination-related knowledge, increased trust in national healthcare guidelines, satisfaction with the national healthcare system, and medical adherence were the strongest predictors of mandatory vaccination support. It is, therefore, vital for national public health authorities to integrate well-organized vaccination campaigns and health promotion interventions targeting both the general population, but also healthcare professionals to ensure that accurate evidence-based information would be disseminated with respect to individuals’ autonomy.

## Figures and Tables

**Table 1:** Sociodemographic characteristics of all participants, overall and by mandatory vaccination support.

Sociodemographic Characteristics	Overall (N = 2140)	Mandatory COVID-19 Vaccination		
		No (N = 1539, 72.2%)	Yes (N = 594, 27.8%)	*p*-Value
**Gender [N^a^ (%)]**				
Female	1323 (61.8)	942 (71.4)	378 (28.6)	0.301 ^h^
Male	817 (38.2)	597 (73.4)	216 (26.6)	
**Mean Age (SD)**	38.2 ± 10.7	37.1 ± 9.9	40.8 ± 12.1	**<0.001 ^i^**
**Geographical area [N^a^ (%)]**				
Nicosia	998 (46.6)	697 (69.9)	300 (30.1)	0.065 ^h^
Limassol	538 (25.1)	406 (75.7)	130 (24.3)	
Larnaca	317 (14.9)	228 (72.6)	86 (27.4)	
Paphos	139 (6.5)	94 (68.1)	44 (31.9)	
Ammochostos	148 (6.9)	114 (77.0)	34 (23.0)	
**Occupation [N^b^ (%)]**				
Private employee	1032 (48.8)	760 (73.8)	270 (26.2)	**<0.001 ^h^**
State employee	474 (22.4)	335 (71.0)	137 (29.0)	
Freelance	231 (10.9)	168 (73.0)	62 (27.0)	
Student	161 (7.6)	114 (71.2)	46 (28.8)	
Unemployed	110 (5.2)	82 (74.5)	28 (25.5)	
Housewife	58 (2.7)	38 (65.5)	20 (34.5)	
Retired	48 (2.4)	18 (37.5)	30 (62.5)	
**Marital status [N^c^ (%)]**				
Married/In cohabitation	1472 (69.7)	1036 (70.6)	432 (29.4)	0.052 ^h^
Unmarried	495 (23.4)	374 (75.7)	120 (24.3)	
Divorced/separated/widowed	145 (6.9)	110 (75.9)	35 (24.1)	
**Underage children living in the household [N^d^ (%)]**				
No	1006 (47.0)	685 (68.4)	317 (31.6)	**<0.001** ^h^
Yes	1133 (53.0)	853 (75.5)	277 (24.5)	
**Healthcare professional [N^e^ (%)]**				
No	2056 (97.3)	1484 (72.3)	567 (27.7)	0.065 ^h^
Yes	57 (2.7)	34 (60.7)	22 (39.3)	
**Education [N^f^ (%)]**				
Up to secondary education	421 (19.9)	326 (77.8)	93 (22.2)	**<0.001 ^h^**
Undergraduate education	961 (45.4)	708 (73.8)	251 (26.2)	
Postgraduate education	734 (34.7)	489 (66.8)	243 (33.2)	
**Annual income [N^g^ (%)]**				
Low (≤€6500)	298 (14.3)	222 (74.7)	75 (25.3)	**<0.001 ^h^**
Moderate (€6500–19,500)	840 (40.4)	654 (78.1)	183 (21.9)	
High (>€19,500)	943 (45.3)	616 (65.4)	326 (34.6)	

Abbreviations: SD; standard deviation; ^a^ N = 2140; ^b^ N = 2114; ^c^ N = 2112; ^d^ N = 2112; ^e^ N = 2107; ^f^ N = 2116; ^g^ N = 2081; ^h^ Differences between mandatory COVID-19 vaccination groups were tested using chi^2^ test; ^I^ Differences between mandatory COVID-19 vaccination groups were tested using *t*-test; Bold values indicate statistically significant associations.

**Table 2:** Information about participants’ health status and attitudes towards healthcare services, overall and by mandatory vaccination support.

Healthcare Services’ Information and Attitudes	Overall (N = 2140)	Mandatory COVID-19 Vaccination		
		No (N = 1539, 72.2%)	Yes (N = 594, 27.8%)	*p*-Value
**Chronic diseases (at least one) [N^a^ (%)]**				
No	1741 (81.7)	1284 (74.0)	452 (26.0)	**<0.001 ^e^**
Yes	390 (18.3)	247 (63.5)	142 (36.5)	
**Use of preventive healthcare services** **(e.g., annual check-up) [N^b^ (%)]**				
Not at all	178 (8.3)	152 (85.9)	25 (14.1)	**<0.001 ^e^**
Little	567 (26.6)	446 (79.1)	118 (20.9)	
Moderate	713 (33.4)	492 (69.1)	220 (30.9)	
Often	563 (26.4)	376 (66.8)	187 (33.2)	
Very often	112 (5.3)	68 (61.3)	43 (38.7)	
**Trust in official guidelines and recommendations by the national healthcare authorities [N^c^ (%)]**				
No trust	619 (29.1)	612 (99.0)	6 (1.0)	**<0.001 ^e^**
Little trust	267 (12.5)	260 (97.7)	6 (2.3)	
Moderate trust	490 (23.0)	394 (80.6)	95 (19.4)	
Strong trust	538 (25.3)	223 (41.6)	313 (58.4)	
Very strong trust	216 (10.1)	41 (19.1)	174 (80.9)	
**Satisfaction with the healthcare system [N^a^ (%)]**				
No satisfied	416 (19.5)	399 (96.4)	15 (3.6)	**<0.001 ^e^**
Little satisfied	488 (22.9)	432 (88.5)	56 (11.5)	
Moderate satisfied	792 (37.2)	537 (68.0)	253 (32.0)	
Very satisfied	394 (18.5)	155 (39.5)	237 (60.5)	
Extremely satisfied	41 (1.9)	10 (24.4)	31 (75.6)	
**Following doctor’s instructions/Medical adherence [N^d^ (%)]**				
Not at all	20 (0.9)	20 (100.0)	0 (0.0)	**<0.001 ^e^**
Little	118 (5.5)	115 (97.5)	3 (2.5)	
Moderate	363 (17.0)	332 (91.7)	30 (8.3)	
Often	1097 (51.4)	774 (70.7)	320 (29.3)	
Very often	536 (25.2)	294 (55.1)	240 (44.9)	

^a^ N = 2131; ^b^ N = 2133; ^c^ N = 2130; ^d^ N = 2134; ^e^ Differences between mandatory COVID-19 vaccination groups were tested using chi^2^ test; Bold values indicate statistically significant associations.

**Table 3:** Participants’ attitudes towards mandatory COVID-19 vaccination, overall and by mandatory vaccination support.

Mandatory COVID-19 Vaccination’s Attitudes	Overall (N = 2140)	Mandatory COVID-19 Vaccination		
		No (N = 1539, 72.2%)	Yes (N = 594, 27.8%)	*p*-Value
**Extent of support of COVID-19 vaccination mandate [N^a^ (%)]**				
Strongly disagree	1146 (53.6)	1142 (99.7)	3 (0.3)	**<0.001 ^g^**
Disagree	202 (9.5)	200 (99.5)	1 (0.5)	
Neither agree nor disagree	213 (10.0)	182 (86.7)	28 (13.3)	
Agree	259 (12.1)	11 (4.3)	247 (95.7)	
Strongly agree	316 (14.8)	2 (0.6)	314 (99.4)	
**Mandatory COVID-19 vaccination is ethically and scientifically justified [N^a^ (%)]**				
Strongly disagree	966 (45.2)	962 (99.7)	3 (0.3)	**<0.001 ^g^**
Disagree	248 (11.6)	244 (98.8)	3 (1.2)	
Neither agree nor disagree	328 (15.4)	243 (74.5)	83 (25.5)	
Agree	322 (15.1)	62 (19.4)	258 (80.6)	
Strongly agree	272 (12.7)	25 (9.2)	247 (90.8)	
**Mandatory COVID-19 vaccination is a policy directed against individual’s personal freedoms [N^b^ (%)]**				
Strongly disagree	266 (12.4)	19 (7.1)	247 (92.9)	**<0.001 ^g^**
Disagree	277 (13.0)	60 (21.8)	215 (78.2)	
Neither agree nor disagree	268 (12.6)	163 (61.3)	103 (38.7)	
Agree	317 (14.9)	290 (91.8)	26 (8.2)	
Strongly agree	1003 (47.1)	999 (99.7)	3 (0.3)	
**Mandatory COVID-19 vaccination violates human rights [N^a^ (%)]**				
Strongly disagree	270 (12.6)	12 (4.4)	258 (95.6)	**<0.001 ^g^**
Disagree	298 (14.0)	59 (19.9)	237 (80.1)	
Neither agree nor disagree	199 (9.3)	119 (60.4)	78 (39.6)	
Agree	275 (12.9)	255 (93.1)	19 (6.9)	
Strongly agree	1094 (51.2)	1091 (99.8)	2 (0.2)	
**Mandatory COVID-19 vaccination is a policy that will reinforce my understanding that the COVID-19 vaccine is necessary [N^c^ (%)]**				
Strongly disagree	982 (46.1)	954 (97.2)	27 (2.8)	**<0.001 ^g^**
Disagree	329 (15.5)	277 (84.4)	51 (15.6)	
Neither agree nor disagree	399 (18.7)	196 (49.5)	200 (50.5)	
Agree	270 (12.7)	80 (29.6)	190 (70.4)	
Strongly agree	148 (7.0)	23 (15.6)	124 (84.4)	
**Mandatory COVID-19 vaccination is a policy that reinforces my perception that vaccine side effects are rare [N^d^ (%)]**				
Strongly disagree	900 (42.6)	870 (96.9)	28 (3.1)	**<0.001 ^g^**
Disagree	374 (17.7)	295 (79.1)	78 (20.9)	
Neither agree nor disagree	460 (21.8)	251 (54.6)	209 (45.4)	
Agree	262 (12.4)	79 (30.4)	181 (69.6)	
Strongly agree	117 (5.5)	22 (18.8)	95 (81.2)	
**Mandatory COVID-19 vaccination is a policy that will reinforce my perception that the vaccine has been studied well [N^e^ (%)]**				
Strongly disagree	946 (44.5)	925 (97.9)	20 (2.1)	**<0.001 ^g^**
Disagree	375 (17.6)	321 (86.1)	52 (13.9)	
Neither agree nor disagree	364 (17.1)	163 (45.0)	199 (55.0)	
Agree	318 (14.9)	97 (30.5)	221 (69.5)	
Strongly agree	126 (5.9)	26 (20.8)	99 (79.2)	
**COVID-19 vaccination should be mandatory for healthcare professionals (e.g., doctors, nurses, etc.) [N^f^ (%)]**				
Strongly disagree	748 (35.0)	744 (99.6)	3 (0.4)	**<0.001 ^g^**
Disagree	255 (11.9)	255 (100.0)	0 (0.0)	
Neither agree nor disagree	281 (13.2)	265 (94.6)	15 (5.4)	
Agree	241 (11.3)	152 (63.3)	88 (36.7)	
Strongly agree	610 (28.6)	120 (19.8)	487 (80.2)	

^a^ N = 2136; ^b^ N = 2131; ^c^ N = 2128; ^d^ N = 2113; ^e^ N = 2129; ^f^ N = 2135; ^g^ Differences between mandatory COVID-19 vaccination groups were tested using chi^2^ test; Bold values indicate statistically significant associations.

**Table 4:** Participants’ general vaccination knowledge, overall and by mandatory vaccination support.

Vaccination Knowledge	Overall (N = 2140)	Mandatory COVID-19 Vaccination		
		No (N = 1539, 72.2%)	Yes (N = 594, 27.8%)	*p*-Value
**Mean knowledge score (SD)**	6.5 ± 2.5	5.9 ± 2.5	8.0 ± 1.9	**<0.001 ^b^**
**Knowledge level [N^a^ (%)]**				
Low (score ≤ 5)	451 (21.1)	425 (94.9)	23 (5.1)	**<0.001 ^c^**
Moderate (score 6–8)	1182 (55.2)	871 (73.8)	309 (26.2)	
High (score ≥ 9)	507 (23.7)	243 (48.1)	262 (51.9)	

Abbreviations: SD, standard deviation; ^a^ N = 2140; ^b^ Differences between mandatory COVID-19 vaccination groups were tested using *t*-test; ^c^ Differences between mandatory COVID-19 vaccination groups were tested using chi^2^ test; Bold values indicate statistically significant associations.

**Table 5:** Hierarchical logistic regression modeling for sociodemographic, vaccination knowledge score, and health status and attitudes towards healthcare services on mandatory vaccination support.

	Model 1		Model 2		Model 3	
Characteristics	OR (95% CI)	*p*-Value	OR (95% CI)	*p*-Value	OR (95% CI)	*p*-Value
**Gender**						
Male	Ref		Ref		Ref	
Female	**1.26 (1.01, 1.57)**	**0.040**	1.08 (0.85, 1.37)	0.510	1.11 (0.83, 1.49)	0.475
**Age (years)**	**1.04 (1.02, 1.05)**	**<0.001**	**1.04 (1.02, 1.05)**	**<0.001**	**1.04 (1.02, 1.05)**	**<0.001**
**Occupation**						
Private employee	Ref		Ref		Ref	
State employee	0.85 (0.65, 1.10)	0.219	0.84 (0.64, 1.12)	0.234	**0.69 (0.49, 0.97)**	**0.035**
Freelance	0.94 (0.66, 1.33)	0.719	0.93 (0.64, 1.34)	0.694	1.07 (0.68, 1.70)	0.760
Unemployed	1.62 (0.90, 2.92)	0.109	1.92 (0.99, 3.72)	0.053	1.79 (0.77, 4.17)	0.179
Student	**3.74 (2.11, 6.62)**	**<0.001**	**2.39 (1.26, 4.52)**	**0.007**	1.49 (0.70, 3.18)	0.302
Housewife	1.98 (1.00, 3.96)	0.051	**2.22 (1.05, 4.70)**	**0.037**	1.83 (0.66, 5.07)	0.242
Retired	1.98 (0.94, 4.17)	0.071	1.75 (0.78, 3.90)	0.172	1.70 (0.57, 5.06)	0.341
**Marital status**						
Married/In cohabitation	Ref		Ref		Ref	
Unmarried	0.82 (0.60, 1.13)	0.230	0.78 (0.56, 1.10)	0.160	0.78 (0.52, 1.18)	0.241
Divorced/separated/widowed	**0.60 (0.39, 0.92)**	**0.019**	0.63 (0.41, 1.01)	0.053	0.77 (0.43, 1.38)	0.373
**Underage children living in the household**						
No	Ref		Ref		Ref	
Yes	**0.62 (0.49, 0.78)**	**<0.001**	**0.58 (0.45, 0.75)**	**<0.001**	**0.69 (0.50, 0.94)**	**0.018**
**Education**						
Up to secondary education	Ref		Ref		Ref	
Undergraduate education	1.28 (0.94, 1.73)	0.116	1.02 (0.74, 1.43)	0.882	1.35 (0.89, 2.05)	0.160
Postgraduate education	**1.83 (1.31, 2.56)**	**<0.001**	1.10 (0.76, 1.58)	0.613	1.04 (0.66, 1.64)	0.853
**Annual income**						
Low	Ref		Ref		Ref	
Moderate	1.41 (0.88, 2.27)	0.146	1.45 (0.86, 2.46)	0.164	1.05 (0.54, 2.02)	0.890
High	**2.38 (1.44, 3.96)**	**0.001**	**1.95 (1.11, 3.43)**	**0.020**	1.42 (0.70, 2.90)	0.330
**General vaccination knowledge score (0–12)**	**-**	**-**	**1.50 (1.42, 1.59)**	**<0.001**	**1.21 (1.13, 1.29)**	**<0.001**
**Chronic diseases**						
No	-	-	-	-	Ref	
Yes	-	-	-	-	1.07 (0.76, 1.51)	0.704
**Use of preventive healthcare services**	-	-	-	-	1.02 (0.89, 1.18)	0.744
**Trust in official guidelines and recommendations by the national healthcare authorities**	-	-	-	-	**3.74 (3.11, 4.49)**	**<0.001**
**Satisfaction with the healthcare system**	-	-	-	-	**1.38 (1.16, 1.65)**	**0.008**
**Following doctor’s instructions/medical adherence**	-	-	-	-	**1.29 (1.03, 1.61)**	**0.024**

Abbreviations: OR, odds ratio; CI, Confidence interval; Model 1: Sociodemographic characteristics on mandatory vaccination support (Yes vs. No); Model 2: Sociodemographic characteristics and vaccination knowledge score on mandatory vaccination support (Yes vs. No); Model 3: Sociodemographic characteristics, vaccination knowledge score, presence of chronic diseases, and attitudes towards healthcare services on mandatory vaccination support (Yes vs. No).

## Data Availability

The datasets used and/or analyzed during the current study available from the corresponding author on reasonable request.

## Supplementary Materials

The following are available online at https://www.mdpi.com/article/10.3390/vaccines10030438/s1, Figure S1. Participants’ reasons for COVID-19 vaccination and reasons for hesitating to get vaccinated against COVID-19. Table S1: Information about participants’ COVID-19 vaccination, overall and by mandatory vaccination support. Table S2: Participants’ general vaccination knowledge, overall and by mandatory vaccination support. Supplementary S1: Electronic consent form and questionnaire in Greek and translated to English.
