# Supplementary material for: Attitudes and Determinants of Mandatory Vaccination against COVID-19 among the General Population of Cyprus: A Nationwide Cross-Sectional Study
Source: Vaccines (Basel). 2022 Mar 13;10(3):438. doi: 10.3390/vaccines10030438 (PMC8953644; doi:10.3390/vaccines10030438)

Figure S1. Participants' reasons for COVID-19 vaccination and reasons for hesitating to get vaccinated against COVID-19.

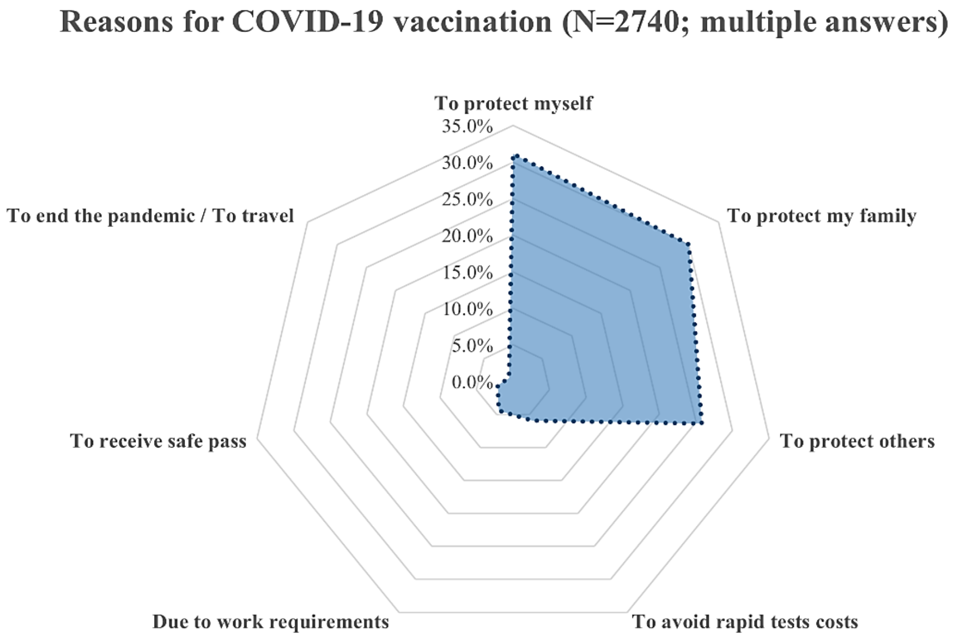

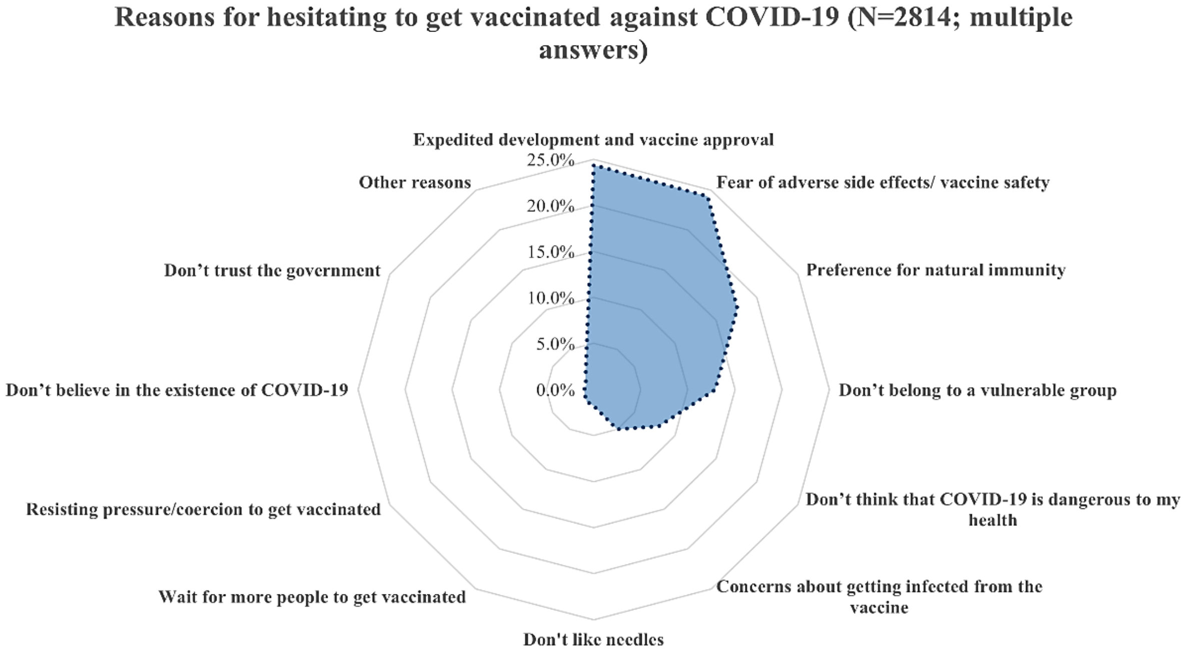

Supplement: Supplementary file 1 [file vaccines-10-00438-s001.zip › Figure S1.pdf]
