# Supplementary material for: Attitudes and Determinants of Mandatory Vaccination against COVID-19 among the General Population of Cyprus: A Nationwide Cross-Sectional Study
Source: Vaccines (Basel). 2022 Mar 13;10(3):438. doi: 10.3390/vaccines10030438 (PMC8953644; doi:10.3390/vaccines10030438)
Supplement: Supplementary file 1 [file vaccines-10-00438-s001.zip › Supplementary file S1.pdf]

Supplementary S1: Electronic consent form and questionnaire in Greek and translated to English. (The original form in Greek is provided below).

UNDERSTANDING THE ATTITUDES AND KNOWLEDGE OF CYPRUS CITIZENS TOWARDS MANDATORY VACCINATION

What is the purpose of this research?

The purpose of this questionnaire is to study the attitudes and perceptions of the general population of Cyprus regarding mandatory vaccination against COVID-19 as well as to assess the level of knowledge about vaccination in general. The questionnaire is anonymous, and your information will remain within the scope of the survey, without the risk of using it for another purpose. The purpose of the research is purely scientific. The results of the research can be compared with similar studies and will contribute to the promotion of public health.

Important information

You will be asked to answer a short anonymous questionnaire. Your participation is anonymous and voluntary, and the time required to complete the questionnaire ranges from 8 to 10 minutes. Please choose the answer that represents your point of view and answer as honestly as possible. All information you provide to us will remain strictly confidential and will not be disclosed to anyone other than the research team. All the information that will be collected will be coded and, after the end of the study, will be destroyed. You can end the completion at any time by closing the browser window. If you do not complete the entire questionnaire, the information you provided on the previous pages will not be recorded and will not be processed.

The online form records only the "timestamp" (i.e., date and time of submission) and no other identifiers (names, IP addresses, etc.). If you would like to withdraw your participation, even after submitting the answers, please note the exact date and time (in the nearest minutes and seconds) you submitted the questionnaire and then contact 22559656 by phone to the lead study coordinator: Dr. Konstantinos Giannakou (K.Giannakou@euc.ac.cy). The present research has not received any funding and has been approved by the National Bioethics Committee of Cyprus (ΕΒΒΚ ΕΠ 2021.01.219).

By clicking YES below, you agree that:

- You have read and understood the aims and objectives of the study
- You have read the instructions and other information and agree to participate voluntarily
- Understand that you can withdraw your answers if you wish
- You agree that the anonymous information you provide will be processed and analyzed
- Fill in and submit your answers only once
- Anonymous information is stored and analyzed

Do you agree to take part in the study?

- o Yes
- o No

SECTION A – DEMOGRAPHICS

A1. What is your gender?
- Male
- Female
- Other (Specify)

A2. What is your age?

____

A3. What is your country of origin?
- Cyprus
- Greece
- Other (Specify)

A4. Which city do you live in?
- Nicosia
- Larnaca
- Limassol
- Ammochostos
- Paphos
- Other (Specify)

A5. What is your profession?
- Private employee
- State employee
- Freelance
- Unemployed
- Housewife
- Student
- Retired
- Other (Specify)

A6. Are you a health professional (eg doctor, nurse, etc.)?
- Yes
- No

A7. If you are a health professional, what profession do you practice?
- Nurse
- Physician
- Dentist
- Pharmacist
- Other (Specify)

A8. What is your marital status?
- Married
- In cohabitation
- Unmarried
- Divorced
- Separated
- Widowed

A9. What is the number of underage children living with you?

____

A10. What is the highest level of education you have completed so far?
- o Primary
- o Gymnasium
- o High School/Technical school
- o College
- o University
- o Postgraduate
- o Doctorate

A11. What is your monthly gross income (euros)?
- o No income
- o Less than 500
- o 501-1000
- o 1001-1500
- o 1501-2000
- o More than 2001

SECTION B - QUESTIONS ABOUT HEALTH AND HEALTH SERVICES

B1. Do you have a chronic illness / disease (at least one, e.g., diabetes, asthma) that you have been diagnosed with by a doctor?
- Yes
- No

B2. How often do you use preventive health care services (e.g., annual check-up)?
- Not at all
- Little
- Moderate
- Often
- Very often

B3. Do you trust the official guidelines and recommendations of the healthcare authorities?
- No trust
- Little trust
- Moderate trust
- Strong trust
- Very strong trust

B4. How satisfied are you with the healthcare system?
- No satisfied
- Little satisfied
- Moderate satisfied
- Very satisfied
- Extremely satisfied

B5. To what extent do you follow the medical instructions given to you by your doctor?
- Not at all
- Little
- Moderate
- Often
- Very often

SECTION C - QUESTIONS ABOUT COVID-19 VACCINATION STATUS

C1. Have you received a vaccine against COVID-19 (if your answer is Yes, go to question C2, if your answer is No, go to C7)?
- o Yes
- o No

C2. How many doses have you taken so far?
- o 1
- o 2
- o 3

C3. Which COVID-19 vaccine did you receive?
- o Pfizer/BioNTech
- o AstraZeneca
- o Moderna
- o Johnson & Johnson
- o Combination
- o Other (Specify)

C4. How likely is it that you will get another dose if requested to do so?
- o Not at all
- o Little
- o Moderate
- o A lot
- o Very much

C5. How much do you think the vaccine helped prevent COVID-19 disease?
- o Not at all
- o Little
- o Moderate
- o A lot
- o Very much

C6. For which of the following reasons you were vaccinated? (more than one reasons can be selected). Proceed to question C9.
- o To protect myself
- o To protect others
- o Because it is strongly encouraged in my workplace
- o To protect my family
- o Because it is provided to me for free
- o Other (Specify)

C7. For which of the following reasons you were not vaccinated? (more than one reasons can be selected).
- o Fear of adverse side effects
- o Expedited development and approval of the vaccine
- o Concerns about getting infected from the vaccine
- o Do not like needles
- o Don’t belong to a vulnerable group
- o Don’t think that COVID-19 is dangerous to my health
- o Preference for natural immunity

- Wait other people to get vaccinated
- Other (Specify)

C8. If you have not received the COVID-19 vaccine to date, do you plan to get it?
- Yes
- No
- I am not sure

C9. Do you belong to a vulnerable group (obese, diabetic, etc.) to whom the COVID-19 vaccine is recommended?
- Yes
- No
- I am not sure

C10. What are your main sources of information on COVID-19 vaccination? (more than one reasons can be selected)
- Internet/social media
- Personal doctor
- Colleagues/friends/family
- Scientific journals
- TV/newspapers/radio
- Conferences/seminars
- Other (Specify)

SECTION D - QUESTIONS ABOUT ATTITUDES AGAINST COVID-19 MANDATORY VACCINATION

D1. Do you think that COVID-19 vaccination should be mandatory on the general population?
- o Yes
- o No

D2. What is your opinion on the introduction of mandatory COVID-19 vaccination on the general population?
- o Strongly disagree
- o Disagree
- o Neither agree nor disagree
- o Agree
- o Strongly agree

D3. Mandatory COVID-19 vaccination is ethically and scientifically justified.
- o Strongly disagree
- o Disagree
- o Neither agree nor disagree
- o Agree
- o Strongly agree

D4. Mandatory COVID-19 vaccination is a policy directed against individual's personal freedoms.
- o Strongly disagree
- o Disagree
- o Neither agree nor disagree
- o Agree
- o Strongly agree

D5. Mandatory COVID-19 vaccination violates human rights.
- o Strongly disagree
- o Disagree
- o Neither agree nor disagree
- o Agree
- o Strongly agree

D6. Mandatory COVID-19 vaccination is a policy that will reinforce my understanding that the COVID-19 vaccine is necessary.
- o Strongly disagree
- o Disagree
- o Neither agree nor disagree
- o Agree
- o Strongly agree

D7. Mandatory COVID-19 vaccination is a policy that reinforces my perception that vaccine side effects are rare.
- o Strongly disagree
- o Disagree
- o Neither agree nor disagree
- o Agree
- o Strongly agree

D8. Mandatory COVID-19 vaccination is a policy that will reinforce my perception that the vaccine has been studied well.

- Strongly disagree
- Disagree
- Neither agree nor disagree
- Agree
- Strongly agree

D9. COVID-19 vaccination should be mandatory for healthcare professionals (e.g., doctors, nurses, etc.).

- Strongly disagree
- Disagree
- Neither agree nor disagree
- Agree
- Strongly agree

SECTION E – GENERAL VACCINATION KNOWLEDGE

E1. The flu is caused by bacteria.
- True
- False
- I do not know

E2. The main route of influenza transmission is through respiratory secretions.
- True
- False
- I do not know

E3. There is a vaccine to prevent cervical cancer.
- True
- False
- I do not know

E4. Some vaccines contain live but attenuated microorganisms.
- True
- False
- I do not know

E5. Most vaccines contain adjuvants to enhance vaccine effectiveness.
- True
- False
- I do not know

E6. The vaccine is a preventive measure.
- True
- False
- I do not know

E7. The transmission of pathogenic microorganisms occurs only through infected individuals.
- True
- False
- I do not know

E8. The measles/mumps/rubella (MMR) vaccine is associated with autism.
- True
- False
- I do not know

E9. If a person is vaccinated, the possibilities of transmitting the disease are higher.
- True
- False
- I do not know

E10. Vaccines are unnecessary, as viruses can be treated with antibiotics.
- True
- False
- I do not know

E11. Vaccination needs to be done for diseases that have been eliminated.
- True
- False
- I do not know

E12. Vaccination increases the incidence of allergies.
- True
- False
- I do not know

SECTION F. RECOMMENDATIONS TO THE RESEARCH GROUP

Write short notes about your impressions of the research topic and any other suggestions or information that may create a new perspective on future research in this field.

END OF QUESTIONNAIRE

Thank you very much for your time and your significant help in implementing this study.

ΚΑΤΑΝΟΗΣΗ ΤΩΝ ΣΤΑΣΕΩΝ ΚΑΙ ΓΝΩΣΕΩΝ ΤΩΝ ΚΥΠΡΙΩΝ ΠΟΛΙΤΩΝ ΑΠΕΝΑΝΤΙ ΣΤΟΝ ΥΠΟΧΡΕΩΤΙΚΟ ΕΜΒΟΛΙΑΣΜΟ

Ποιος είναι ο σκοπός της έρευνας;
Το παρόν ερωτηματολόγιο έχει σκοπό την μελέτη διερεύνησης στάσεων και αντιλήψεων του γενικού πληθυσμού της Κύπρου ως προς τον υποχρεωτικό εμβολιασμό κατά του COVID-19 καθώς και την εκτίμηση του επιπέδου γνώσεων περί του εμβολιασμού γενικότερα. Το ερωτηματολόγιο είναι ανώνυμο και τα στοιχεία σας θα παραμείνουν εντός του πλαισίου της έρευνας, χωρίς να υπάρχει κίνδυνος χρήσης τους για άλλο σκοπό. Ο σκοπός της έρευνας είναι αποκλειστικά επιστημονικός. Τα αποτελέσματα της ερευνάς θα μπορούν να συγκριθούν με παρόμοιες μελέτες και θα συνεισφέρουν στην προαγωγή της Δημοσίας Υγειάς.

Σημαντικές πληροφορίες
Θα σας ζητηθεί να απαντήσετε σε ένα σύντομο ανώνυμο ερωτηματολόγιο. Η συμμετοχή σας είναι ανώνυμη και εθελοντική και ο απαιτούμενος χρόνος για τη συμπλήρωση του ερωτηματολογίου κυμαίνεται από 8 έως 10 λεπτά. Παρακαλώ επιλέξτε την απάντηση που αντιπροσωπεύει την άποψή σας και απαντήστε όσο το δυνατόν πιο ειλικρινά. Όλες οι πληροφορίες που θα μας δώσετε θα παραμείνουν αυστηρά εμπιστευτικές και δεν θα γίνουν γνωστές σε κανένα άλλο άτομο, εκτός από το προσωπικό της έρευνας. Όλες οι πληροφορίες που θα συλλεχθούν, θα κωδικοποιηθούν και, μετά το τέλος της μελέτης θα καταστραφούν. Μπορείτε να τερματίσετε τη συμπλήρωση ανά πάσα στιγμή κλείνοντας το παράθυρο του προγράμματος περιήγησης. Εάν δεν συμπληρώσετε όλο το ερωτηματολόγιο, οι πληροφορίες που δώσατε στις προηγούμενες σελίδες δε θα καταγραφούν και δεν θα υποστούν επεξεργασία.

Η ηλεκτρονική φόρμα καταγράφει μόνο το "timestamp" (δηλαδή, ημερομηνία και ώρα υποβολής) και κανένα άλλο αναγνωριστικό στοιχείο (ονόματα, διευθύνσεις IP, κ.λπ.). Εάν θέλετε να αποσύρετε τη συμμετοχή σας, ακόμα και αφού υποβάλετε τις απαντήσεις, παρακαλώ να σημειώσετε την ακριβή ημερομηνία και ώρα (στα κοντινότερα λεπτά και δευτερόλεπτα) που υποβάλατε το ερωτηματολόγιο και έπειτα επικοινωνήστε τηλεφωνικώς στο 22559656 στον υπεύθυνο συντονιστή της μελέτης: Δρ. Κωνσταντίνο Γιαννακού (K.Giannakou@euc.ac.cy). Η παρούσα έρευνα δεν έχει λάβει οποιαδήποτε χρηματοδότηση και έχει εγκριθεί από την Εθνική Επιτροπή Βιοηθικής Κύπρου (ΕΒΒΚ ΕΠ 2021.01.219).

Πατώντας ΝΑΙ πιο κάτω, συμφωνείτε ότι:

- Έχετε διαβάσει και έχετε κατανοήσει τους σκοπούς και στόχους της μελέτης
- Έχετε διαβάσει τις οδηγίες και άλλες πληροφορίες και συμφωνείτε να συμμετάσχετε εθελοντικά
- Καταλαβαίνετε ότι μπορείτε να αποσύρετε τις απαντήσεις σας εάν θέλετε
- Συμφωνείτε ότι οι ανώνυμες πληροφορίες που παρέχετε θα τύχουν επεξεργασίας και ανάλυσης
- Συμπληρώνετε και υποβάλλετε τις απαντήσεις σας μόνο μία φορά
- Οι ανώνυμες πληροφορίες αποθηκεύονται και αναλύονται

Συναινείτε να λάβετε μέρος στην μελέτη;

o Ναι
o Όχι

ΜΕΡΟΣ Α – ΔΗΜΟΓΡΑΦΙΚΑ ΣΤΟΙΧΕΙΑ

Α1. Ποιο είναι το φύλο σας;
- Άνδρας
- Γυναίκα
- Άλλο (Προσδιορίστε)

Α2. Ποια είναι η ηλικία σας;

____

Α3. Ποια είναι η χώρα καταγωγής σας;
- Κύπρος
- Ελλάδα
- Άλλο (Προσδιορίστε)

Α4. Σε ποια επαρχία κατοικείτε;
- Λευκωσία
- Λάρνακα
- Λεμεσός
- Αμμόχωστος
- Πάφος
- Άλλο (Προσδιορίστε)

Α5. Ποιο είναι το επάγγελμά σας;
- Ιδιωτικός υπάλληλος
- Δημόσιος υπάλληλος
- Ελεύθερος/η επαγγελματίας
- Άνεργος/η
- Οικιακά
- Φοιτητής/τρια
- Συνταξιούχος/χα
- Άλλο (Προσδιορίστε)

Α6. Είστε επαγγελματίας υγείας (π.χ. γιατρός, νοσηλευτής, κλπ..);
- Ναι
- Όχι

Α7. Αν είστε επαγγελματίας υγείας, τι επάγγελμα εξασκείτε;
- Νοσηλευτής/τρια
- Γιατρός
- Οδοντίατρος
- Φαρμακοποιός
- Άλλο (Προσδιορίστε)

Α8. Ποια η οικογενειακή σας κατάσταση;
- Έγγαμος-η
- Σε συμβίωση
- Άγαμος-η
- Διαζευγμένος-η
- Σε διάσταση
- Χήρος-α

A9. Ποιος είναι ο αριθμός ανήλικων παιδιών που διαμένουν μαζί σας;

____

A10. Ποιο είναι το υψηλότερο επίπεδο εκπαίδευσης που έχετε ολοκληρώσει μέχρι τώρα;
- Δημοτικό
- Γυμνάσιο
- Λύκειο/Τεχνική Σχολή
- Κολλέγιο
- Πανεπιστήμιο
- Μεταπτυχιακό
- Διδακτορικό

A11. Ποιο είναι το μηνιαίο σας ακαθάριστο εισόδημα (ευρώ);
- Δεν έχω εισόδημα
- Λιγότερο από 500
- 501-1000
- 1001-1500
- 1501-2000
- Περισσότερο από 2001

ΜΕΡΟΣ Β - ΕΡΩΤΗΣΕΙΣ ΣΧΕΤΙΚΑ ΜΕ ΤΗΝ ΥΓΕΙΑ ΚΑΙ ΤΙΣ ΥΠΗΡΕΣΙΕΣ ΥΓΕΙΑΣ

Β1. Πάσχετε από κάποια χρόνια πάθηση/νόσημα (τουλάχιστον ένα, π.χ. διαβήτης, άσθμα) για την οποία έχετε διαγνωστεί από κάποιο γιατρό;
- Ναι
- Όχι

Β2. Πόσο συχνά χρησιμοποιείτε προληπτικές υπηρεσίες υγειονομικής περίθαλψης (π.χ. ετήσιος έλεγχος/check-up);
- Καθόλου
- Λίγο
- Μέτρια
- Συχνά
- Πολύ συχνά

Β3. Έχετε εμπιστοσύνη στις επίσημες οδηγίες και συστάσεις των αρχών υγειονομικής περίθαλψης για θέματα υγειονομικής περίθαλψης (π.χ. εμβολιασμός);
- Δεν έχω εμπιστοσύνη
- Έχω λίγη εμπιστοσύνη
- Έχω μερική εμπιστοσύνη
- Έχω πολλή εμπιστοσύνη
- Έχω πάρα πολλή εμπιστοσύνη

Β4. Πόσο ικανοποιημένος/η είστε από το σύστημα υγειονομικής περίθαλψης;
- Καθόλου
- Λίγο ικανοποιημένος/η
- Μερικώς ικανοποιημένος/η
- Πολύ ικανοποιημένος/η
- Πάρα πολύ ικανοποιημένος/η

Β5. Σε τι βαθμό ακολουθείτε τις ιατρικές οδηγίες που σας δίνονται από τον θεράποντα ιατρό σας;
- Καθόλου
- Λίγο
- Μέτρια
- Πολύ
- Πάρα πολύ

ΜΕΡΟΣ Γ - ΕΡΩΤΗΣΕΙΣ ΣΧΕΤΙΚΑ ΜΕ ΤΗΝ ΚΑΤΑΣΤΑΣΗ ΕΜΒΟΛΙΑΣΜΟΥ ΚΑΤΑ ΤΟΥ COVID-19

Γ1. Έχετε λάβει εμβόλιο κατά του COVID-19 (αν η απάντηση σας είναι Ναι, προχωρήστε στην ερώτηση Γ2, αν η απάντηση σας είναι Όχι, προχωρήστε στην Γ7);
- Ναι
- Όχι

Γ2. Πόσες δόσεις έχετε κάνει μέχρι σήμερα;
- 1
- 2
- 3

Γ3. Ποιο εμβόλιο κατά του COVID-19 λάβατε;
- Pfizer/BioNTech
- AstraZeneca
- Moderna
- Johnson & Johnson
- Συνδυασμός
- Άλλο (Προσδιορίστε)

Γ4. Πόσο πιθανό είναι να κάνετε και άλλη δόση αν προαιρετικά ζητηθεί;
- Καθόλου
- Λίγο
- Μέτρια
- Πολύ
- Πάρα πολύ

Γ5. Πόσο πιστεύετε συνέβαλε το εμβόλιο στην αποφυγή νόσησης από τον COVID-19;
- Καθόλου
- Λίγο
- Μέτρια
- Πολύ
- Πάρα πολύ

Γ6. Για ποιους από τους παρακάτω λόγους εμβολιαστήκατε; (Σημειώστε ότι ισχύει, ένα ή περισσότερα). Προχωρήστε στην ερώτηση Γ9.
- Για να προστατεύσω τον εαυτό μου
- Για να προστατεύσω τους άλλους
- Γιατί ενθαρρύνεται έντονα στο χώρο της εργασίας μου
- Για να προστατεύσω την οικογένειά μου
- Γιατί μου παρέχεται δωρεάν
- Άλλο (Προσδιορίστε)

Γ7. Για ποιους από τους παρακάτω λόγους δεν εμβολιαστήκατε; (Σημειώστε ότι ισχύει, ένα ή περισσότερα)
- Φόβος για ανεπιθύμητες ενέργειες
- Αμφισβήτηση ελέγχων ποιότητας – δημιουργήθηκε πολύ γρήγορα
- Φόβος ότι αν εμβολιαστώ, θα νοσήσω
- Δεν μου αρέσουν οι ενέσεις (πόνος και δυσφορία)
- Δεν θεωρώ ότι ανήκω σε ομάδα υψηλού κινδύνου
- Δεν θεωρώ την συγκεκριμένη ασθένεια επικίνδυνη

- Υποστηρίζω την φυσική ανοσοποίηση
- Θα αναμένω να εμβολιαστούν άλλοι πρώτα
- Άλλο (Προσδιορίστε)

Γ8. Εάν δεν έχετε λάβει το εμβόλιο κατά του COVID-19 μέχρι σήμερα, προγραμματίζετε να εμβολιαστείτε;
- Ναι
- Όχι
- Δεν είμαι σίγουρος/η

Γ9. Ανήκετε σε κάποια ευάλωτη ομάδα (παχύσαρκος/η, σακχαρώδη διαβήτη κ.α.) στους οποίους προτείνεται ο εμβολιασμός κατά του COVID-19;
- Ναι
- Όχι
- Δεν είμαι σίγουρος/η

Γ10. Ποιες είναι οι κύριες πηγές ενημέρωσής σας για τις εξελίξεις σε θέματα σχετικά με τον εμβολιασμό κατά του COVID-19; (Σημειώστε ότι ισχύει, ένα ή περισσότερα)
- Διαδίκτυο/Μέσα κοινωνικής δικτύωσης
- Προσωπικός ιατρός
- Συνάδελφοι/φίλοι/οικογένεια
- Επιστημονικά περιοδικά
- Τηλεόραση και άλλα μέσα μαζικής ενημέρωσης
- Εκπαιδευτικές εκδηλώσεις/σεμινάρια
- Άλλο (Προσδιορίστε)

ΜΕΡΟΣ Δ - ΕΡΩΤΗΣΕΙΣ ΣΧΕΤΙΚΑ ΜΕ ΤΙΣ ΣΤΑΣΕΙΣ ΣΑΣ ΕΝΑΝΤΙ ΤΟΥ ΥΠΟΧΡΕΩΤΙΚΟΥ ΕΜΒΟΛΙΑΣΜΟΥ ΚΑΤΑ ΤΟΥ COVID-19

Δ1. Πιστεύετε ότι ο εμβολιασμός κατά του COVID-19 πρέπει να είναι υποχρεωτικός στον γενικό πληθυσμό;
- Ναι
- Όχι

Δ2. Ποια είναι η γνώμη σας για την εισαγωγή του υποχρεωτικού εμβολιασμού κατά του COVID-19 στον γενικό πληθυσμό.
- Διαφωνώ απόλυτα
- Διαφωνώ
- Ούτε συμφωνώ, ούτε διαφωνώ
- Συμφωνώ
- Συμφωνώ απόλυτα

Δ3. Η υποχρεωτική διάθεση των εμβολίων κατά του COVID-19 είναι ηθικά και επιστημονικά δικαιολογημένη.
- Διαφωνώ απόλυτα
- Διαφωνώ
- Ούτε συμφωνώ, ούτε διαφωνώ
- Συμφωνώ
- Συμφωνώ απόλυτα

Δ4. Η υποχρεωτική διάθεση των εμβολίων κατά του COVID-19 είναι μια πολιτική που στρέφεται κατά των προσωπικών ελευθεριών του ατόμου.
- Διαφωνώ απόλυτα
- Διαφωνώ
- Ούτε συμφωνώ, ούτε διαφωνώ
- Συμφωνώ
- Συμφωνώ απόλυτα

Δ5. Πιστεύετε ότι ο υποχρεωτικός εμβολιασμός καταπατά τα ανθρώπινα δικαιώματα.
- Διαφωνώ απόλυτα
- Διαφωνώ
- Ούτε συμφωνώ, ούτε διαφωνώ
- Συμφωνώ
- Συμφωνώ απόλυτα

Δ6. Η υποχρεωτική διάθεση των εμβολίων κατά του COVID-19 είναι πολιτική που θα ενισχύσει την αντίληψη μου ότι το εμβόλιο κατά του COVID-19 είναι απαραίτητο.
- Διαφωνώ απόλυτα
- Διαφωνώ
- Ούτε συμφωνώ, ούτε διαφωνώ
- Συμφωνώ
- Συμφωνώ απόλυτα

Δ7. Η υποχρεωτική διάθεση των εμβολίων κατά του COVID-19 είναι πολιτική που θα ενισχύσει την αντίληψη μου ότι οι ανεπιθύμητες ενέργειες του εμβολίου είναι σπάνιες.
- Διαφωνώ απόλυτα
- Διαφωνώ

- Ούτε συμφωνώ, ούτε διαφωνώ
- Συμφωνώ
- Συμφωνώ απόλυτα

Δ8. Η υποχρεωτική διάθεση των εμβολίων κατά του COVID-19 είναι πολιτική που θα ενισχύσει την αντίληψη μου ότι το εμβόλιο έχει μελετηθεί αρκετά.
- Διαφωνώ απόλυτα
- Διαφωνώ
- Ούτε συμφωνώ, ούτε διαφωνώ
- Συμφωνώ
- Συμφωνώ απόλυτα

Δ9. Οι επαγγελματίες υγείας (π.χ. γιατροί, νοσηλευτές κλπ.) είναι αναγκαίο να εμβολιάζονται υποχρεωτικά κατά του COVID-19.
- Διαφωνώ απόλυτα
- Διαφωνώ
- Ούτε συμφωνώ, ούτε διαφωνώ
- Συμφωνώ
- Συμφωνώ απόλυτα

ΜΕΡΟΣ Ε - ΓΝΩΣΕΙΣ ΓΙΑ ΤΟΝ ΕΜΒΟΛΙΑΣΜΟ

Ε1. Η γρίπη προκαλείται από βακτήρια;
- Σωστό
- Λάθος
- Δεν γνωρίζω

Ε2. Η κύρια οδός μετάδοσης της γρίπης είναι μέσω αναπνευστικών εκκρίσεων;
- Σωστό
- Λάθος
- Δεν γνωρίζω

Ε3. Υπάρχει εμβόλιο για πρόληψη του καρκίνου του τραχήλου της μήτρας;
- Σωστό
- Λάθος
- Δεν γνωρίζω

Ε4. Κάποια εμβόλια περιέχουν ζωντανούς αλλά εξασθενημένους μικροοργανισμούς;
- Σωστό
- Λάθος
- Δεν γνωρίζω

Ε5. Τα περισσότερα εμβόλια περιέχουν ανοσοενισχυτικά για ενίσχυση του αποτελεσματικότητας του εμβολίου;
- Σωστό
- Λάθος
- Δεν γνωρίζω

Ε6. Το εμβόλιο αποτελεί μέσο προφύλαξης;
- Σωστό
- Λάθος
- Δεν γνωρίζω

Ε7. Η μετάδοση των παθογόνων μικροοργανισμών γίνεται μέσω μολυσμένων ατόμων;
- Σωστό
- Λάθος
- Δεν γνωρίζω

Ε8. Το εμβόλιο για ιλαρά/ερυθρά/παρωτίτιδα (MMR) σχετίζεται με τον αυτισμό;
- Σωστό
- Λάθος
- Δεν γνωρίζω

Ε9. Αν ένα άτομο είναι εμβολιασμένο, οι πιθανότητες να μεταδώσει την ασθένεια είναι περισσότερες;
- Σωστό
- Λάθος
- Δεν γνωρίζω

Ε10. Τα εμβόλια είναι περιττά, καθώς οι ιοί μπορούν να αντιμετωπιστούν με αντιβιοτικά;
- Σωστό
- Λάθος

- Δεν γνωρίζω

Ε11. Χρειάζεται να γίνεται εμβολιασμός για νοσήματα που έχουν εξαφανιστεί;
- Σωστό
- Λάθος
- Δεν γνωρίζω

Ε12. Ο εμβολιασμός αυξάνει την εμφάνιση αλλεργιών.
- Σωστό
- Λάθος
- Δεν γνωρίζω

ΜΕΡΟΣ ΣΤ. ΕΙΣΗΓΗΣΕΙΣ ΠΡΟΣ ΤΗΝ ΕΡΕΥΝΗΤΙΚΗ ΟΜΑΔΑ

Γράψε σύντομες σημειώσεις για τις εντυπώσεις σου σχετικά με το ερευνητικό θέμα και οποιαδήποτε άλλη εισήγηση ή πληροφορία, η οποία μπορεί να δημιουργήσει μία νέα οπτική για την έρευνα σε αυτό το πεδίο στο μέλλον.

ΤΕΛΟΣ ΕΡΩΤΗΜΑΤΟΛΟΓΙΟΥ

Σας ευχαριστούμε πολύ για το χρόνο που διαθέσατε και τη σημαντική βοήθειά σας για την υλοποίηση της παρούσας μελέτης.
