# Supplementary material for: Attitudes and Determinants of Mandatory Vaccination against COVID-19 among the General Population of Cyprus: A Nationwide Cross-Sectional Study
Source: Vaccines (Basel). 2022 Mar 13;10(3):438. doi: 10.3390/vaccines10030438 (PMC8953644; doi:10.3390/vaccines10030438)
Supplement: Supplementary file 1 [file vaccines-10-00438-s001.zip › Table S1_v1.2.pdf]

Table S1. Information about participants' COVID-19 vaccination, overall and by mandatory vaccination support.

**COVID-19 vaccination**	**Overall** (N = 2140)	**Mandatory COVID-19 vaccination**		
		**No** (N = 1539)	**Yes** (N = 594)	**p-value**
**Vaccination status** [N[a] (%)]				
No	974 (46.0)	967 (99.4)	6 (0.6)	**<0.01**[h]
Yes	1143 (54.0)	555 (48.8)	583 (51.2)	
**Number of doses** [N[b] (%)]				
1	77 (6.6)	61 (79.2)	16 (20.8)	**<0.01**[h]
2	760 (65.6)	405 (53.5)	352 (46.5)	
3	322 (27.8)	99 (30.9)	221 (69.1)	
**Type of COVID-19 vaccine** [N[c] (%)]				
Pfizer	792 (68.3)	377 (47.7)	413 (52.3)	**0.001**[h]
Moderna	145 (12.5)	81 (56.2)	63 (43.8)	
Astra Zeneca	119 (10.3)	47 (40.2)	70 (59.8)	
Johnson & Johnson	64 (5.5)	45 (70.3)	19 (26.7)	
Sputnik	3 (0.3)	1 (33.3)	2 (66.7)	
Combination	37 (3.1)	14 (37.8)	23 (62.2)	
**Intention to receive another dose if requested** [N[d] (%)]				
Not at all	226 (17.8)	220 (97.3)	6 (2.7)	**<0.01**[h]
Little	74 (5.8)	64 (90.1)	7 (9.9)	
Moderate	147 (11.6)	116 (78.9)	31 (21.1)	
A lot	299 (23.5)	131 (44.0)	167 (56.0)	
Very much	525 (41.3)	146 (27.9)	378 (72.1)	
**Belief that vaccine helped to prevent COVID-19 disease?** [N[e] (%)]				
Not at all	238 (17.6)	232 (97.5)	6 (2.5)	**<0.01**[h]
Little	131 (9.7)	125 (96.2)	5 (3.8)	
Moderate	229 (16.9)	161 (70.6)	67 (29.4)	
A lot	384 (28.4)	166 (43.3)	217 (56.7)	
Very much	370 (27.4)	74 (20.1)	294 (79.9)	
**If you have not received the COVID-19 vaccine to date, do you plan to receive it?** [N[f] (%)]				
I do not know	174 (17.3)	172 (98.8)	2 (1.2)	**<0.01**[h]
No	805 (80.2)	800 (99.5)	4 (0.5)	
Yes	25 (2.5)	18 (72.0)	7 (28.0)	
**They belong to a vulnerable group (diabetic, immunosuppressed, etc.) to whom vaccination is recommended** [N[g] (%)]				
I do not know	94 (4.5)	66 (70.2)	28 (29.8)	**<0.01**[h]
No	1764 (84.3)	1310 (74.5)	449 (25.5)	
Yes	235 (11.2)	142 (60.7)	92 (39.3)	

[a]N=2117; [b]N=1159; [c]N=1160; [d]N=1271; [e]N=1352; [e]N=1004; [g]N=2093; [h]Differences between mandatory COVID-19 vaccination groups were tested using chi^2 test; Bold values indicate statistically significant associations.
