# Supplementary material for: Attitudes and Determinants of Mandatory Vaccination against COVID-19 among the General Population of Cyprus: A Nationwide Cross-Sectional Study
Source: Vaccines (Basel). 2022 Mar 13;10(3):438. doi: 10.3390/vaccines10030438 (PMC8953644; doi:10.3390/vaccines10030438)
Supplement: Supplementary file 1 [file vaccines-10-00438-s001.zip › Table S2_v1.2.pdf]

Table S2. Participants' general vaccination knowledge overall and by mandatory vaccination support.

	**Overall** (N = 2140)	**Mandatory COVID-19 vaccination**		
		**No** (N = 1539)	**Yes** (N = 594)	**p-value**
**The flu is caused by bacteria** [N[a] (%)]				
True	669 (31.6)	508 (75.9)	161 (24.1)	**0.019**[j]
I do not know	532 (25.2)	367 (69.2)	163 (30.8)	
False	913 (43.2)	642 (70.6)	267 (29.4)	
**The main route of influenza transmission is through respiratory secretions** [N[b] (%)]				
True	1766 (83.5)	1247 (70.7)	517 (29.3)	**0.014**[j]
I do not know	238 (11.2)	180 (76.6)	55 (23.4)	
False	112 (5.3)	90 (81.1)	21 (18.9)	
**There is a vaccine to prevent cervical cancer** [N[c] (%)]				
True	1826 (86.0)	1277 (70.1)	545 (29.9)	**<0.01**[j]
I do not know	236 (11.1)	198 (84.6)	36 (15.4)	
False	61 (2.9)	50 (82.0)	11 (18.0)	
**Some vaccines contain live but attenuated microorganisms** [N[b] (%)]				
True	1562 (73.8)	1136 (72.9)	423 (27.1)	0.177[j]
I do not know	434 (20.5)	305 (70.6)	127 (29.4)	
False	120 (5.7)	78 (65.5)	41 (34.5)	
**Most vaccines contain adjuvants to enhance vaccine effectiveness** [N[d] (%)]				
True	701 (33.2)	490 (70.0)	210 (30.0)	**0.011**[j]
I do not know	1341 (63.5)	965 (72.2)	371 (27.8)	
False	69 (3.3)	60 (87.0)	9 (13.0)	
**The vaccine is a preventive measure** [N[e] (%)]				
True	1699 (80.9)	1117 (65.9)	577 (34.1)	**<0.01**[j]
I do not know	175 (8.3)	172 (98.3)	3 (1.7)	
False	225 (10.8)	211 (94.2)	13 (5.8)	
**The transmission of pathogenic microorganisms occurs only through infected individuals** [N[f] (%)]				
True	1381 (65.8)	946 (68.7)	431 (31.3)	**<0.01**[j]
I do not know	519 (24.7)	404 (78.1)	113 (21.9)	
False	198 (9.5)	150 (75.8)	48 (24.2)	
**The measles/mumps/rubella (MMR) vaccine is associated with autism** [N[g] (%)]				
True	213 (10.1)	197 (92.5)	16 (7.5)	**<0.01**[j]
I do not know	1049 (49.8)	838 (80.1)	208 (19.9)	
False	844 (40.1)	474 (56.4)	367 (43.6)	
**If a person is vaccinated, the possibilities of transmitting the disease are higher** [N[e] (%)]				
True	341 (16.3)	321 (94.4)	19 (5.6)	**<0.01**[j]
I do not know	418 (19.9)	395 (94.7)	22 (5.3)	
False	1340 (63.8)	785 (58.8)	551 (41.2)	
**Vaccines are unnecessary, as viruses can be treated with antibiotics** [N[h] (%)]				
True	235 (11.1)	231 (98.7)	3 (1.3)	**<0.01**[j]
I do not know	300 (14.2)	278 (93.0)	21 (7.0)	
False	1574 (74.7)	1001 (63.8)	569 (36.2)	
**Vaccination needs to be done for diseases that have been eliminated** [N[i] (%)]				
True	943 (44.7)	561 (59.7)	379 (40.3)	**<0.01**[j]
I do not know	638 (30.2)	504 (79.4)	131 (20.6)	
False	531 (25.1)	450 (84.7)	81 (15.3)	
**Vaccination increases the incidence of allergies** [N[d] (%)]				
True	529 (25.1)	502 (95.1)	26 (4.9)	**<0.01**[j]
I do not know	999 (47.3)	754 (75.8)	241 (24.2)	
False	583 (27.6)	257 (44.2)	325 (55.8)	

[a]N=2114; [b]N=2116; [c]N=2123; [d]N=2111; [e]N=2099; [f]N=2098; [g]N=2106; [h]N=2109; [i]N=2112; [j]Differences between mandatory COVID-19 vaccination groups were tested using chi2 test; Bold values indicate statistically significant associations.
